# Non-native species in the vascular flora of highlands and mountains of Iceland

**DOI:** 10.7717/peerj.1559

**Published:** 2016-01-11

**Authors:** Pawel Wasowicz

**Affiliations:** Icelandic Institute of Natural History, Akureyri, Iceland

**Keywords:** Alien flora, Iceland, Highland, Arctic, invasive species, Mountain flora, Tourism, Non-native flora

## Abstract

The highlands and mountains of Iceland are one of the largest remaining wilderness areas in Europe. This study aimed to provide comprehensive and up-to-date data on non-native plant species in these areas and to answer the following questions: (1) How many non-native vascular plant species inhabit highland and mountainous environments in Iceland? (2) Do temporal trends in the immigration of alien species to Iceland differ between highland and lowland areas? (3) Does the incidence of alien species in the disturbed and undisturbed areas within Icelandic highlands differ? (4) Does the spread of non-native species in Iceland proceed from lowlands to highlands? and (5) Can we detect hot-spots in the distribution of non-native taxa within the highlands? Overall, 16 non-native vascular plant species were detected, including 11 casuals and 5 naturalized taxa (1 invasive). Results showed that temporal trends in alien species immigration to highland and lowland areas are similar, but it is clear that the process of colonization of highland areas is still in its initial phase. Non-native plants tended to occur close to man-made infrastructure and buildings including huts, shelters, roads etc. Analysis of spatio-temporal patterns showed that the spread within highland areas is a second step in non-native plant colonization in Iceland. Several statically significant hot spots of alien plant occurrences were identified using the Getis-Ord Gi* statistic and these were linked to human disturbance. This research suggests that human-mediated dispersal is the main driving force increasing the risk of invasion in Iceland’s highlands and mountain areas.

## Introduction

While it is well known that the average proportion of non-native species in polar regions is very low (Elven et al., 2011; Ellis, Antill & Kreft, 2012; Alsos, Ware & Elven, 2015), the total number of alien plant species in the local floras may vary considerably. In the Arctic, the number of both casual and naturalized aliens grows rapidly from the polar Arctic desert zone (where alien species are absent) towards low Arctic floras (southern Greenland, northern Scandinavia, and Iceland), where the proportion of non-native taxa is higher (Lassuy and Lewis, 2013; Wasowicz, Przedpelska-Wasowicz & Kristinsson, 2013). In fact, the non-native plants introduced in the Arctic during centuries of human activity have significantly influenced both the composition of local floras as well as native vegetation patterns (Elven et al., 2011; Alsos, Ware & Elven, 2015).

The beginning of human impact on the vascular flora of Iceland dates back to the ninth century, when the island was first reached by Norse settlers migrating across the North Atlantic. The vegetation of Iceland has changed significantly since that time of settlement. Growing anthropogenic pressure and climatic changes during the “Little Ice Age” (1600–1900 AD) led to significant alteration of the vegetation cover, mainly due to erosion and desertification (Hallsdottir and Caseldine, 2005). However, until recently the composition of vascular flora had remained only mildly affected with changes primarily due the harsh climatic conditions and isolation by the surrounding ocean (Wasowicz, Przedpelska-Wasowicz & Kristinsson, 2013).

The latest research on the non-native flora of Iceland has shown that there are significant differences in the composition of the local floras in Iceland (Wasowicz, Przedpelska-Wasowicz & Kristinsson, 2013). The results showed that while lowland floras host a great number of imported taxa, highland and mountain areas seem to be almost free of alien species. The highlands and mountains of Iceland—defined here as areas located above 400 m above sea level and that account for approximately 40% of the country’s terrain—are some of the most pristine environments in Europe due to their remoteness and harsh climate (Einarsson, 1984). The Central Highlands are considered the largest territories in Europe south of the Arctic Circle that have never been permanently settled by humans (Vésteinsson, 1998). Recent research has revealed the key role these areas play in maintaining natural plant distribution patterns for many native species in Iceland (Wasowicz et al., 2014).

There are two main factors responsible for the low rate of colonization of these areas by non-native species: the low frequency of human-mediated plant dispersal and the harsh climate (see Supplemental Information 1), which is characterized by low temperatures (mean annual temperature below 0 °C), lasting snow cover, and minimal duration of the growing season (approximately 2 months on average) (Thorhallsdóttir, 1996; Thórhallsdóttir, 1998). However, these two constraints are rapidly changing due to an unprecedented increase in human activity within the highlands (Sæþórsdóttir & Saarinen, 2015) as well as ongoing climate warming (Wasowicz, Przedpelska-Wasowicz & Kristinsson, 2013). The establishment and spread of alien species in the Icelandic highlands may be expected to escalate even more dramatically in the near future, which may lead to major environmental change. The combined effects of the introduction of non-native species as well as climate change on biodiversity and ecosystem function can be significant and include, among other things, alteration of the community composition and structure, alterations in trophic pathways and interactions, and changes in native species distribution, habitat structure, as well as in the evolutionary processes of native species (Mooney and Cleland, 2001; Hellmann et al., 2008; Rahel and Olden, 2008; Lassuy and Lewis, 2013).

Future changes in the flora of highland areas of Iceland will be best understood only if measured against a credible baseline. Given the fact that no attempts have been made to summarize existing data concerning non-native plant species in the Icelandic highlands, the present study aimed to answer the following questions:

1. How many non-native vascular plant species inhabit highland and mountainous environments in Iceland?
2. Do temporal trends in the immigration of alien species to Iceland differ between highland and lowland areas?
3. Does the incidence of alien species in disturbed and undisturbed areas within the Icelandic highlands differ?
4. Does the spread of non-native species in Iceland proceed from lowlands to highlands?
5. Can we detect hot-spots in the distribution of non-native taxa within the Icelandic highlands?

## Materials and Methods

### Definitions used

This study is focused on non-native plant species as defined by Pyšek et al. (2004), as taxa whose presence in a given area is due to intentional or unintentional human involvement, or that have arrived there without human intervention from an area where they are alien. Non-native species are further subdivided into two categories: casual and naturalized species. Casual species are alien plants that may flourish and even reproduce occasionally beyond cultivation in an area, but that eventually die out because they do not form self-sustaining populations and instead rely on repeated introductions for their persistence. Naturalized species, on the other hand, are defined as alien plants with self-sustaining populations for at least ten years without direct human intervention (or in spite of human intervention), but rather by recruitment from seed or ramets (tillers, tubers, bulbs, fragments, etc.). Invasive alien species are included under naturalized taxa, and defined as taxa that form self-replacing populations over many life cycles, produce reproductive offspring (often in very large numbers at considerable distances from the parent and/or site of introduction), and have the potential to spread over long distances (Pyšek et al., 2012).

### Plant distribution data

Data were obtained from the Icelandic Institute of Natural History. The Institute has the largest repository of biodiversity data in Iceland, with over 500,000 georeferenced records of plant species distributions. Only records of non-native taxa (Wasowicz, Przedpelska-Wasowicz & Kristinsson, 2013) were considered for the study. Overall, 9,396 records collected between 1840 and 2014 were examined, including vouchered specimens deposited in AMNH and ICEL herbaria, field observations, and literature data records (Supplemental Information 2).

### Spatial analyses

All georeferenced data from the database were converted into shapefiles using QGIS (QGIS Development Team, 2015). Elevation data were retrieved from a digital elevation model of Iceland (20 meters per pixel) downloaded from The Icelandic Geoportal (http://gatt.lmi.is). Elevation (in meters) was then assigned to each data point using the QGIS point sampling tool. A database was developed that contained georeferenced species occurrences linked with elevation data. This database was queried to identify records with an altitude of at least 400 m above sea level (124 in total; Supplemental Information 2).

### Checklist

A checklist of non-native taxa was developed, summarizing information on taxonomy, time of residence, naturalization status, biogeographical affinities, and life form (Raunkiær, 1935). Native distribution of alien taxa was recorded at the continental scale.

### Spatio-temporal trends in colonization

The year of initial observation for each species record was retrieved from the database. The cumulative number of species introduced and the number of observations were plotted against time. Curves were plotted in SigmaPlot (Systat Software, San Jose, CA, USA) using locally weighted regression - LOESS (Cleveland, 1979), using a sampling proportion of 0.1 and polynomial degree set to 1. Distribution maps were plotted in QGIS (QGIS Development Team, 2015).

### Hot spot analysis

Hot Spot Analysis (Mitchell, 2005) was employed to identify statistically significant spatial clusters within the analyzed dataset. This analysis uses the Getis-Ord Gi* statistic (Ord and Getis, 1995) for each feature (species occurrence) in the data set. The analysis works by comparing each occurrence within the context of neighboring occurrences to determine whether a cluster of species observations (a feature with high value) is statistically significant (surrounded by other features with high values). The local sum of a feature and its neighbors is proportionally compared to the total sum of all features. Statistically significant Z-scores result when the local sum is much different than the expected local sum, indicating that difference is too large to be the result of random chance. The Getis-Ord local statistic is given by the following equation:

$$G_i^* = {{\sum\limits_{j = 1}^n {{w_{i,j}}} {x_{i,j}} - \bar X\sum\limits_{j = 1}^n {{w_{i,j}}} } \over {S\sqrt {{{(n\sum\limits_{j = 1}^n {w_{i,j}^2} - {{(\sum\limits_{j = 1}^n {{w_{i,j}}} )}^2})} \over {n - 1}}} }}$$

where *x*_i_ is attribute value for feature *j*, *w_i_*, *j* is the spatial weight between feature *i* and *j*, *n* is equal to the total number of features and

$$\bar X = {{\sum\limits_{j = 1}^n {{x_j}} } \over n}$$

$$S = \sqrt {{{\sum\limits_{j = 1}^n {x_j^2} } \over n} - {{(\bar X)}^2}} $$

The $G_i^*$ statistic calculated in this way is a Z-score (no further calculations are needed). In case of statistically positive Z-scores, the higher the Z-score is, the more intense the clustering of high values - indicating a statistically significant hot spot.

A 10 × 10 kilometer grid used in Iceland to record and map plant occurrences (Wasowicz et al., 2014) was used in the hot-spot analysis. Only polygons containing areas located above 400 meters above sea level were taken into account. The number of alien species records within each polygon was aggregated using Spatial Joint tool, and the main analysis was performed using the Hot Spot Analysis tool. All calculations were carried out using ArcGis 10.2. (ESRI, 2013) using fixed distance band as a conceptualization of spatial relationship and the euclidean distance. Z-score values for each 10 × 10 polygon were visualized on a map.

### Spatial patterns

The presence or absence of non-native plant species in each grid cell (10 × 10 km) was determined using QGIS software (QGIS Development Team, 2015) as well as the presence/absence of human-made infrastructure, such as buildings and roads. A chi-square test was used to evaluate whether disturbed and undisturbed grid cells differ in terms of alien species occurrence. Distance from species occurrence point to the nearest man-made object was also calculated using QGIS software and compared between the analyzed taxa using Kruskal-Wallis test and Nemeyri *post-hoc* test (Pohlert, 2014). Statistical analysis was performed using the computing environment R (R Development Core Team, 2013). GIS layers with the data concerning the spatial distribution of man-made objects and roads were downloaded from The Icelandic Geoportal (http://gatt.lmi.is).

## Results and Discussion

### The number of non-native taxa and their origin

Overall, 16 non-native plant taxa were recorded in the Icelandic highlands and mountain areas between 1840 and 2014 (Table 1). According to the criteria proposed by Pyšek et al. (2004), 11 taxa (69% of total non-native flora) were classified as casual aliens, while five taxa (the remainig 31% of non-native flora) were classified as naturalized. Based on the criteria by Pyšek et al. (2012), only *Lupinus nootkatensis* can be classified as an invasive plant. Comparison of the geographic origin of non-native plant taxa showed that most have a European origin, constituting 66% of all non-native flora in the highlands and 49.2% of all non-native plants in Iceland (Wasowicz, Przedpelska-Wasowicz & Kristinsson, 2013). Also, taxa of Asian and Northern American origin scored high in both in the highlands and the country as a whole. In the highlands, non-native plants from Asia accounted for 56% all alien flora, while 37% of them were from North America. The percentage of non-native plants from North America in the Icelandic highlands is significantly greater than that seen for the entire country (8.9%) (Wasowicz, Przedpelska-Wasowicz & Kristinsson, 2013). This is due to the fact that several non-native species from Northern America have been deliberately introduced into the highlands, becoming naturalized at a faster rate than other non-native species. Plants such as *Lupinus nootkatensis*, *Deschampsia caespitosa* subsp. *beringensis* and *Salix alaxensis* are good examples of this. All three species have their native range in northern and north western parts of North America, where they reach the sub-alpine and alpine zones as well as the arctic part of North America, and inhabit environments similar to those found in the Icelandic highlands (Argus, Eckenwalder & Kiger, 2002; Douglas, Meidinger & Pojar, 1999; Douglas, Meidinger & Pojar, 2000; Douglas, Meidinger & Pojar, 2001). Given the high level of environmental matching, these species are most likely to spread quickly and effectively in the highlands.

**Table 1:** Checklist of non-native vascular plants in highland and mountain areas of Iceland. First record–year of the first record of the species within highland areas, year of first record in Iceland was given in brackets; Last record–year of the last record of the species within highland areas; Naturalization status given for highland areas, status in Iceland (Wasowicz, Przedpelska-Wasowicz & Kristinsson, 2013) was given in brackets; Life form–assigned according to (Raunkiær, 1935); Origin–geographic origin of the species; N–total number of examined records.

	Species	First record	Last record	Naturalization status	Life form	Origin	N
1	*Alnus viridis* ssp. *sinuata*	2005 (1996)	2005	CAS (NAT)	N	NAm, Asi	2
2	*Alopecurus pratensis*	1963 (1902)	2007	**NAT** (NAT)	H	Eu, Asi	15
3	*Avenula pubescens*	1978 (1937)	1978	CAS (NAT)	H	Eu, Asi	1
4	*Claytonia sibirica*	2004 (2004)	2004	CAS (CAS)	H	Asi, NAm	1
5	*Deschampsia cespitosa* subsp. *beringensis*	1996 (1986)	2012	**NAT** (NAT)	H	NAm, Asi	23
6	*Lamium amplexicaule*	1988 (1893)	1988	CAS (NAT)	T	Eu	1
7	*Lappula squarrosa*	1888 (1888)	1888	CAS (CAS)	H	Eu, Asi	1
8	*Lepidotheca suaveolens*	1969 (1902)	1999	CAS (NAT)	T	Eu, NAm, Asi	3
9	*Lolium perenne*	1981 (1909)	1981	CAS (CAS)	H	Eu, Asi	1
10	*Lupinus nootkatensis*	1980 (1967)	2014	**INV** (INV)	H	NAm	44
11	*Myosotis scorpioides*	1978 (1929)	1980	CAS (NAT)	H	Eu, Asi	2
12	*Phleum pratense*	1935 (1887)	2010	**NAT** (NAT)	H	Eu	22
13	*Rheum rhabarbarum*	1996 (1912)	1996	CAS (CAS)	G	cult	1
14	*Salix alaxensis*	2011 (1998)	2011	**NAT** (NAT)	P	NAm	1
15	*Sinapis arvensis*	1937 (1892)	1937	CAS (CAS)	T	Eu	1
16	*Stellaria graminea*	1946 (1861)	2000	CAS (NAT)	H	Eu	5

**Abbreviations:**

CAS, casual; NAT, naturalized; INV, invasive; G, geophyte; H, hemicryptophyte; T, therophyte; N, nano-phanerophytes; P, phanerophytes; Eu, Europe; Asi, Asia; NAm, North America; cult, cultivated taxon.

Currently, Alaska lupine (*L. nootkatensis*) is the most widespread and invasive non-native plant in the Icelandic highlands. Due to the presence of nitrogen-fixing bacteria in the roots, the species can increase the availability of this nutrient in the soil, which otherwise is low, as in most of the Arctic environments (Dowdall et al., 2005; Forbes & Jefferies, 1999; Liška and Soldán, 2004). Lupine-induced change in nutrient content may further exacerbate the problem of alien species invasion. Several experimental studies have shown that alien species are favored when nutrients are added to the nutrient-poor soil (Aber et al., 1989; Chapin, Vitousek & Van Cleve, 1986). Given the fact that native species are adapted to nutrient-poor conditions in Arctic environments (Dowdall et al., 2005; Liška and Soldán, 2004), they may possess poorer competitive qualities than non-native species when faced with the increase in soil nutrient availability (Chapin, Vitousek & Van Cleve, 1986).

### Temporal trends in alien species immigration to Iceland and to highland areas

**Figure 1:**
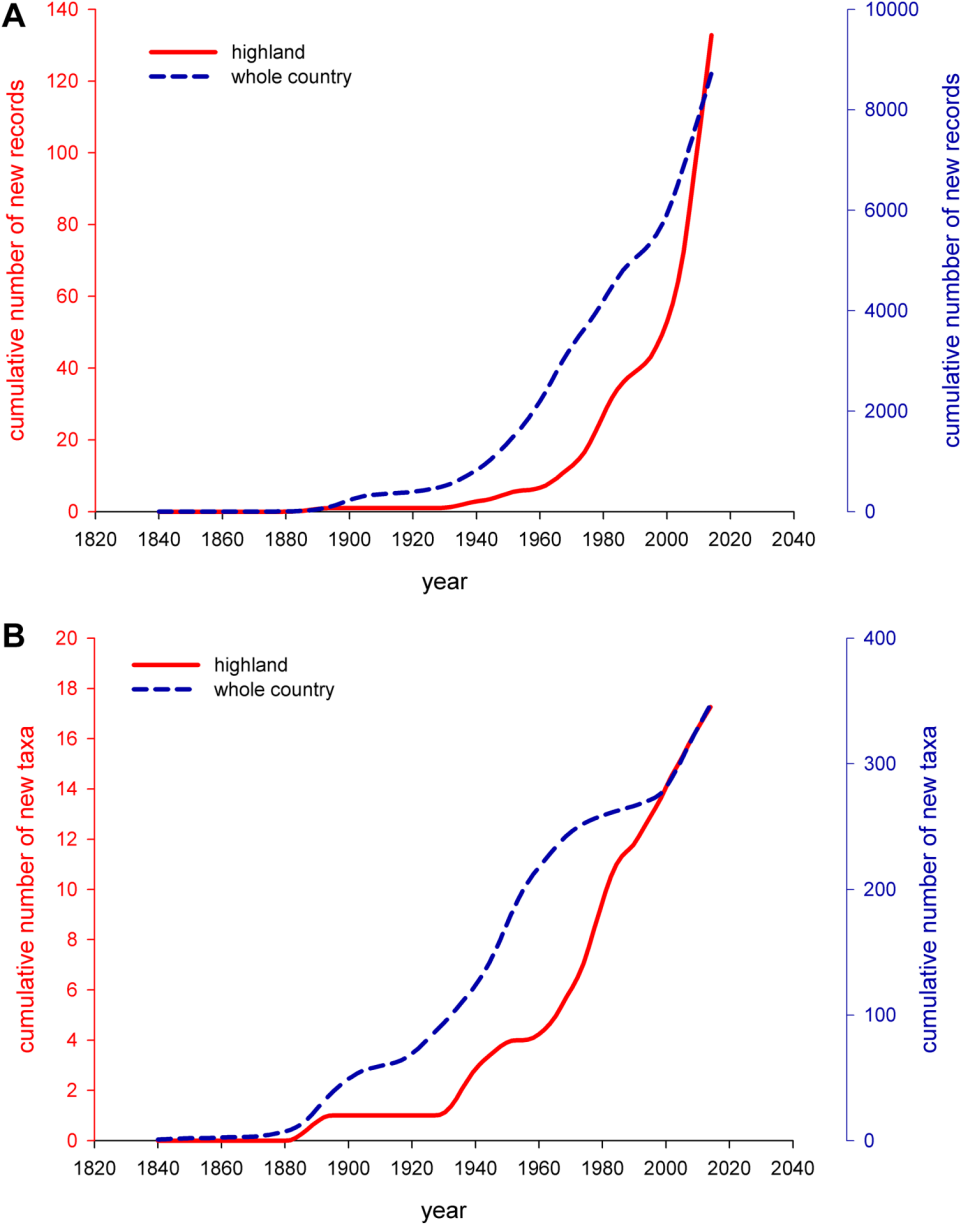
LOESS curves showing dynamics and temporal trends in non-native flora of highland and mountain areas of Iceland (1840–2014). Red y-axis corresponds to the highland areas, blue y-axis to the whole country. (**A**): number of observations, (**B**): number of species. Cumulative numbers were calculated on the basis of *per annum* new taxa records/observations.

The cumulative number of first taxa records was plotted against time to examine temporal trends in alien taxa immigration to the highlands and mountain areas. The analysis showed that the trend recorded for the highlands and mountain areas is very similar to the overall trend of non-native species immigration to Iceland (Fig. 1A). It seems, however, that a steady, linear increase in the number of non-native taxa records started much later in highlands than in Iceland as a whole (Fig. 1A). The same pattern is clearly visible when the number of observations of non-native plant taxa is taken into account (Fig. 1B). A clear growth trend is seen in the highlands a few decades after it started in the lowlands or Iceland as a whole. The curve for the highland areas is much steeper than the curve plotted for the entire country (Fig. 1B). General trends characterizing non-native plant colonization show that this process is still in its initial phase. A relatively low number of non-native plant species was recorded in the highland areas when compared to the rich alien flora in the lowlands (16 vs. over 300 taxa) (Wasowicz, Przedpelska-Wasowicz & Kristinsson, 2013). This suggests that further colonization may occur, particularly if climatic constraints are significantly reduced or even removed by climate change, which has been suggested by recent modelling experiments (Wasowicz, Przedpelska-Wasowicz & Kristinsson, 2013). A sharp increase in the number of species observations after 1960, may be indicative that construction of the first large hydropower plants in the central highlands in the mid-1960s contributed to an increase alien plant colonization. The construction of these hydropower plants involved significant improvements in road infrastructure, making some areas more accessible (Sæþórsdóttir & Saarinen, 2015).

### Do human-disturbed and undisturbed areas differ in terms of alien species occurrence?

**Figure 2:**
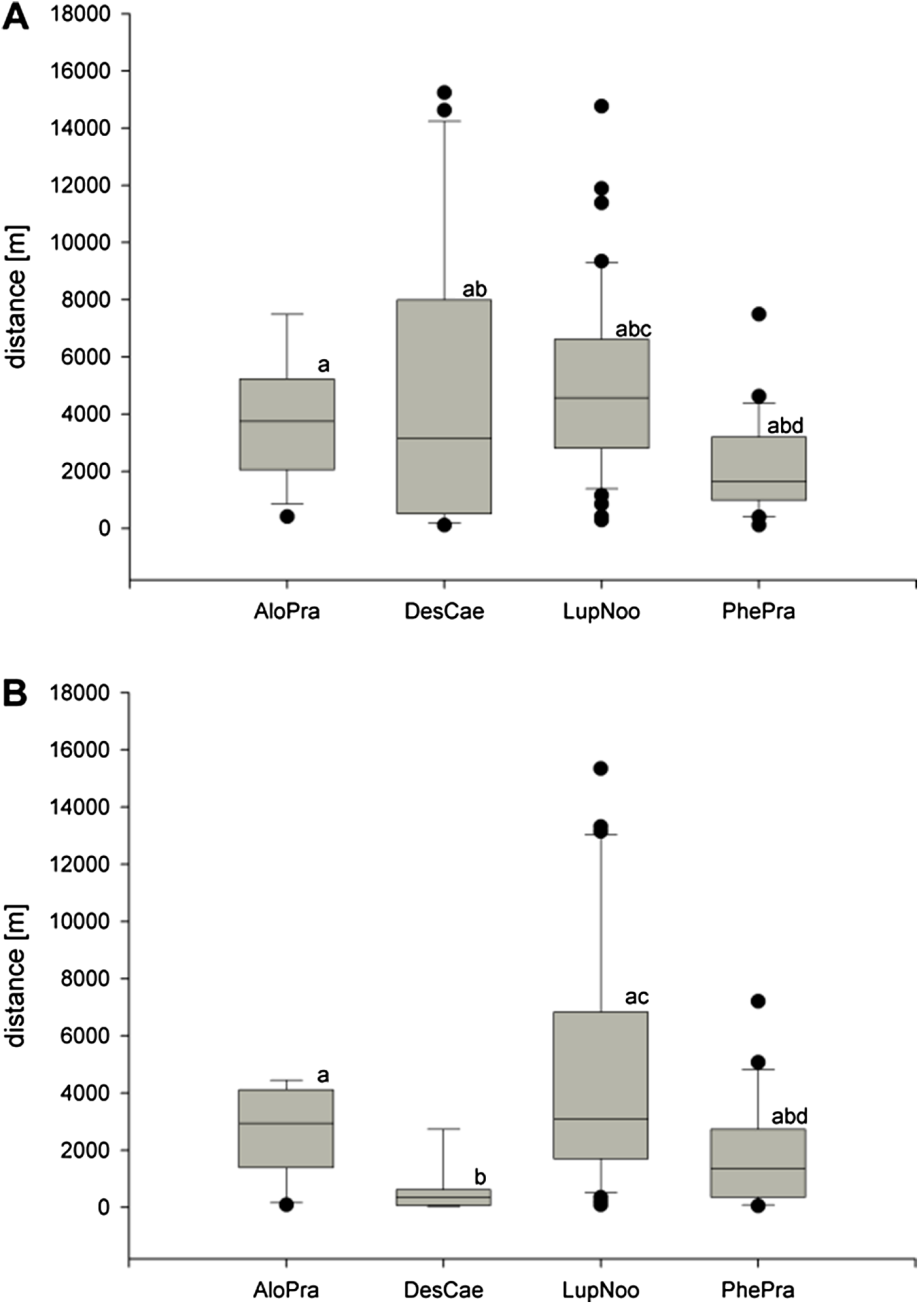
Distances from the place of occurrence to the closest (A) man-made objects (B) roads. Statistical significance (denoted by letters) was tested using Kruskal-Wallis and Nemeyri *post-hoc* test. AloPra, *Alopecurus pratensis*; DesCae, *Deschampsia caespitosa* subsp. *beringensis*; LupNoo, *Lupinus nootkatensis*; PhePra, *Phleum pratense*.

The chi-square test confirmed that human-disturbed (with the presence of man-made objects such as roads, huts, etc.) and undisturbed areas (10 × 10 km polygons) differ in terms of the occurrence of non-native species (χ^2^ = 26.3301, df = 1, p<0.001). All investigated non-native species showed clear tendency to occur within approximately 5 km from man-made objects (huts, small buildings, etc.) and roads (Figs. 2A and 2B). Investigated taxa differed in terms of their distance from the closest man-made object (Kruskal-Wallis test chi squared = 14.2016, df = 3, p = 0.002643). *Phleum pratense* was found to occur in the closest proximity to such infrastructure (median distance: 1361 m, Fig. 2A). Analyzed species also differed in their proximity to roads (Kruskal-Wallis test chi-squared = 37.6923, df = 3, p<0.001). *Deschampsia caespitosa* subsp. *beringensis* was found to occur in the closest proximity to roads and tracks (median distance: 353 m, Fig. 2B).

The distribution and spread of non-native plants is the result of complex interactions between species and environmental conditions (Richardson, Williams & Hobbs, 1994; Thomas & Moloney, 2013). Apart from adaptations to effective spread and reproduction, invasion potential is also determined by ecosystem conditions and human-mediated disturbance (Beans, Kilkenny & Galloway, 2012; Catford et al., 2012). Human activities, as well as the density of human occupancy, have positive associations with invasive plant distributions (Decker et al., 2012). The results presented here support these findings further by showing that areas disturbed by humans differ in terms of the occurrence of non-native taxa from the areas that have not been disturbed. The presence of an association between human disturbance and the distribution of non-native plants was already suggested from the Arctic (Elven et al., 2011) but the present study is the first one to show evidence for this relationship. The Icelandic highlands offer a unique perspective for research focused on the interaction between human disturbance and colonization by non-native taxa for several reasons: (1) the area has never been settled by humans; (2) the impact of non-native taxa on local flora is still minimal, with a very limited number of alien species that are not widespread; and (3) there are processes underway (see below) that are likely to change the pattern of human disturbance. Given that the present study creates a baseline for research on non-native plants in Icelandic highlands, forthcoming changes in local flora and species distribution recorded in this area can be identified more easily and analyzed to greater accuracy than in many other regions of the world.

### Does the spread of non-native species in Iceland proceed from lowlands to highlands?

**Figure 3:**
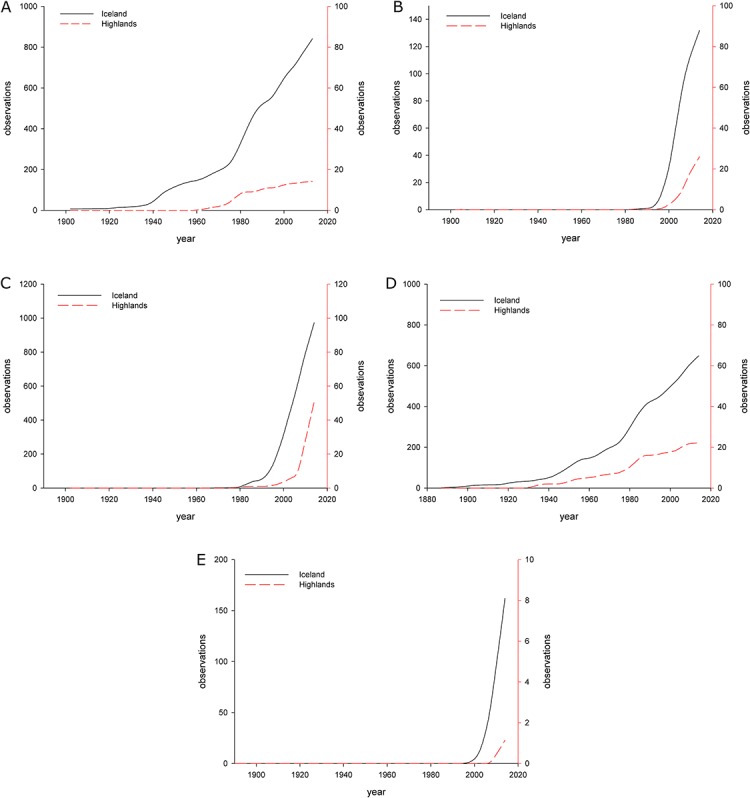
LOESS curves showing the cumulative number of observations (unique records) in Iceland and in highland areas. Dark y-axis corresponds to the whole country, red y-axis to the highland areas. (A) *Alopecurus pratnesis* (B) *Deschampsia caespitosa* subsp. *beringensis* (C) *Lupinus nootkatensis* (D) *Phleum pratense* (E) *Salix alaxensis*.

Taxa naturalized in highland environments (*Alopecurus pratensis*, *Deschampsia caespitosa* subsp. *beringensis*, *Lupinus nootkatensis*, *Phleum pratense* and *Salix alaxensis*) were analyzed to test the hypothesis that the colonization of highland habitats in Iceland can be considered as a “second step” in the process of colonization of the island by non-native taxa. Results showed that species naturalized within highland areas were first well-established in lowland areas, and only then started to spread within the highlands (Fig. 3). This clear spatial and temporal trend was present in all analyzed cases (4 taxa). Furthermore, all taxa naturalized in the highlands and mountain areas have been recorded as naturalized in the lowlands (Wasowicz, Przedpelska-Wasowicz & Kristinsson, 2013), and most of the casual taxa in the highlands are already naturalized in the lowlands (Wasowicz, Przedpelska-Wasowicz & Kristinsson, 2013). There is no evidence available so far showing a different direction of the naturalization process (i.e. a species naturalized in highlands and spreading down into lowlands), and so it follows that the colonization of the highland environments in Iceland is a second step in the process of naturalization of species within the country. Climate is one potential factor that may explain the low rate of colonization success among non-native plants in the highlands. With very few exceptions, most of the highlands and mountain areas in Iceland have a mean July temperature of less than 10 °C (Einarsson, 1984), and thus can be treated as arctic areas based on climatic criteria (Przybylak, 2002). Modeling experiments carried out recently have shown that these unfavorable conditions with low temperature and very short vegetation period restrict the growth of many species in the Icelandic highlands and mountain areas, including native (Wasowicz et al., 2014) and non-native taxa (Wasowicz, Przedpelska-Wasowicz & Kristinsson, 2013).

### Can we identify hot-spots in the distribution of non-native taxa?

**Figure 4:**
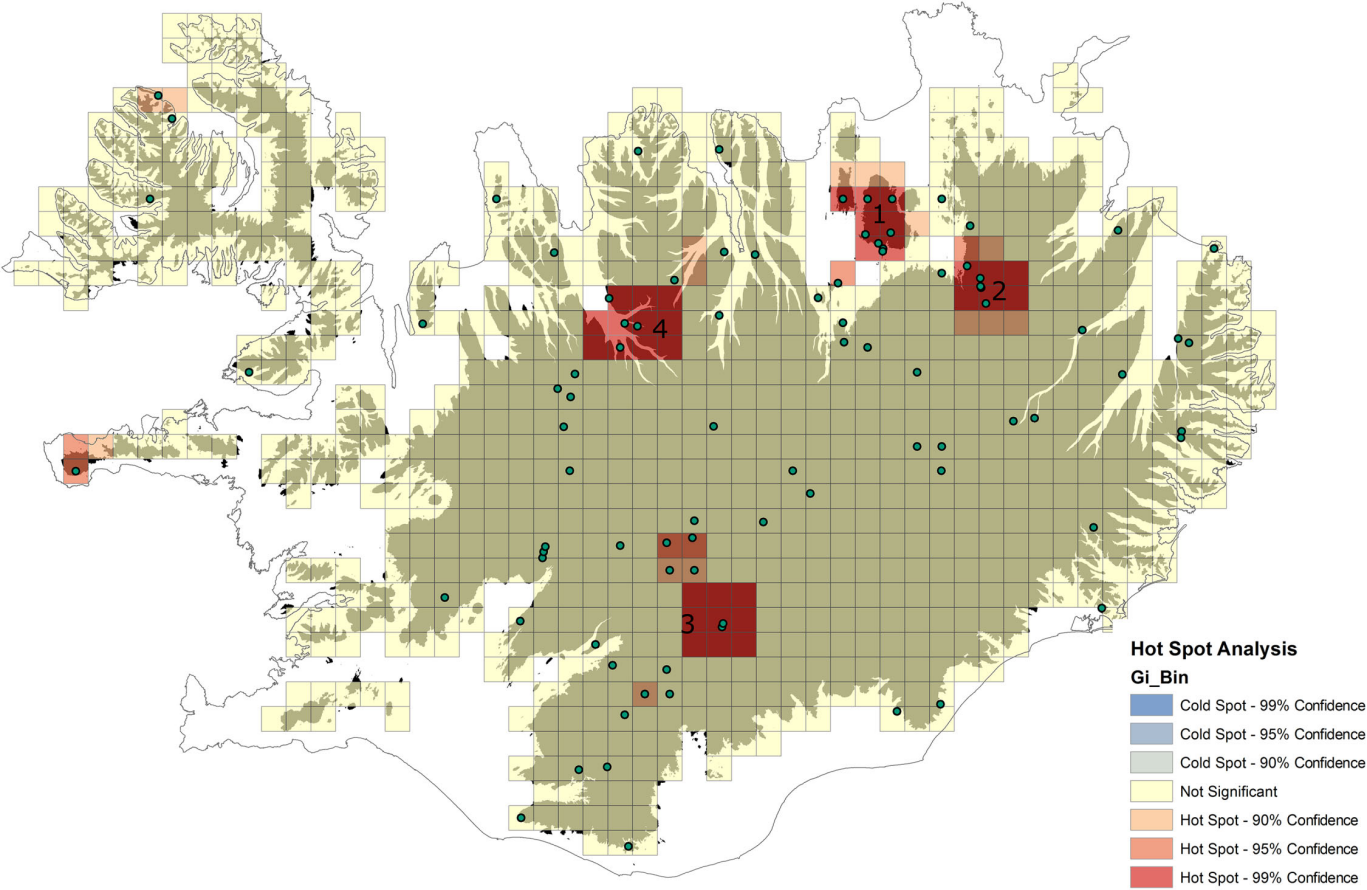
Results of Hot Spot Analysis showing statistically significant spatial clusters of non-native taxa within highland areas in Iceland (color scale—see details on the figure): 1: around Mývatn lake including Rejkjahlið, Námafjall, Krafla volcano; 2: Viðidalur and Vegaskarð, 3: areas W from Vatnajökull glacier (including i.e. Landmannalaugar and Jökulheimar); 4: highland areas bordering with the S part of Skagafjörður. Occurrences of non-native plant taxa were marked with green points.

The Hot Spot Analysis was used to identify statistically significant spatial clusters with a high number of non-native species records. The analysis showed that at least four main spatial clusters can be identified (Fig. 4): (1) the areas around Mývatn lake including Rejkjahlið, Namafjall, Krafla volcano and north of it, (2) the area of Viðidalur and Vegaskarð, (3) the areas west from the Vatnajökull glacier (including Landmannalaugar and Jökulheimar), as well as (4) the highland areas bordering with the southern part of Skagafjörður in northern Iceland. Apart from these clusters, the general tendency is that most of the non-native plants occur within the Central Highland, while highland and mountain areas in other parts of the country still remain almost free of non-native taxa (Fig. 4).

Fairly accessible highland areas close to Mývatn Lake (north-eastern Iceland) have been strongly influenced by naturalized alien plant species. The climatic conditions in the northeastern part of the country allowed human settlement and farming activities at greater than 400 meters above sea level, facilitating the introduction of non-native plants. In contrast, areas located at greater than 400 meters above sea level in other parts of Iceland (e.g. the Western Fjords and Eastern Fjords) appear to be almost free of non-native plant species. This pattern of spatial occurrence can be explained by human-mediated dispersal. A closer examination of places with very high numbers of non-native species records shows that they are mostly found in areas with tourist attractions such as hot springs (e.g. Hveravellir, Laugafell) and areas with geothermal activity (e.g. Reykjahlíð, Námafjall and Krafla volcano), as well as near hiking huts and shelters along the highlands (e.g. Jökulheimar).

The study seems to confirm that human-mediated dispersal along a road network is one of the most important factors contributing to plant dispersal (von der Lippe et al., 2013). It is clear that all occurrences in spatial cluster covering areas of Viðidalur and Vegaskarð are related either to propagule transport along the road network or to the direct spread of species used for restoration purposes along roads (e.g. *D.caespitosa* susbp. *beringensis*).

Only one spatial cluster identified during the present study (areas around southern part of Skagafjörður) seems to depart from what was said above. This cluster contains mostly occurrences of grass species commonly used in Iceland as fodder - *Alpoecurus pratensis* and *Phleum pratense* - and possibly suggests that penetration of agricultural species into the highlands within this area might be more dynamic than in other regions.

### Future of the flora of Icelandic highlands

In recent years, Arctic wilderness environments have become a major tourist attraction, while the Central Highlands in Iceland are considered one of the largest remaining wilderness areas in Europe (Sæþórsdóttir & Saarinen, 2015). The increase in international visitors coming to Iceland is particularly high, where the number of tourists has grown from 72,600 in 1982 to approximately 1 million in 2014 (Sæþórsdóttir & Saarinen, 2015). It is estimated that about one-third of tourists visit the Central Highlands (Icelandic Tourist Board, 2013). These values suggest that the influx of propagules carried on the clothing, gear, and vehicles of visitors to the Central Highland is very likely higher than ever before and will probably continue to grow with increasing tourism; recent studies in Antarctica and the Arctic further support the importance of this type of plant propagule transfer to spreading non-native species (Whinam, Chilcott & Bergstrom, 2005; Lee and Chown, 2009; Ware et al., 2012; Chown et al., 2012; Huiskes et al., 2014). It is highly likely that increased propagule pressure will contribute to increased secondary invasions by existing non-native species through facilitated seed transport along road networks (von der Lippe et al., 2013), as well as through the arrival of new alien species brought by tourists from lowland areas and abroad. An example of this is *Digitaria ischaemum* (Poaceae), which seems to have spread to thermal areas in southern Iceland *via* visitors’ hiking shoes. Future actions to facilitate travel through the central highlands (e.g. construction of new road tracks or improvement of existing routes), will inevitably increase the number of visitors, leading to a greater inflow of seeds and other plant propagules of non-native taxa and therefore a higher risk of invasion. Increased colonization due to increased propagule transfer, may be further facilitated by climatic changes. It has been shown that climatic niche of many non-native species in Iceland will increase dramatically in forthcoming decades (Wasowicz, Przedpelska-Wasowicz & Kristinsson, 2013).

## Conclusions

1. Sixteen non-native vascular plant taxa were recorded in the Icelandic highlands and mountains.
2. Temporal trends in alien species immigration to lowland and highland areas of Iceland are similar, but the process of colonization of highland areas is still in its initial phase.
3. Non-native plants tend to occur close to all types of man-made infrastructure and buildings including huts, shelters, and road networks.
4. The spread of non-native species in Iceland proceeds from lowlands to highlands.
5. Several statically significant hot-spots of alien plant occurrences can be identified and are linked to human disturbance.

## Additional Information and Declarations

### Data Deposition

The following information was supplied regarding data availability: All the data used during this study can be found in Supplemental Information 2.
