# Supplementary figures and images for: Non-native species in the vascular flora of highlands and mountains of Iceland
Source: PeerJ. 2016 Jan 11;4:e1559. doi: 10.7717/peerj.1559 (PMC4736984; doi:10.7717/peerj.1559)

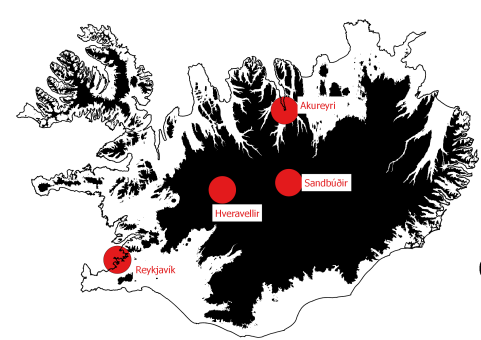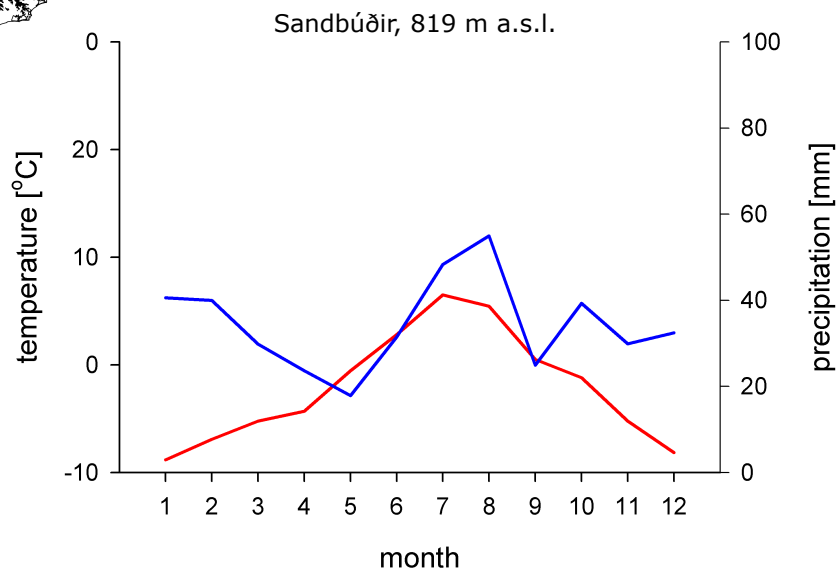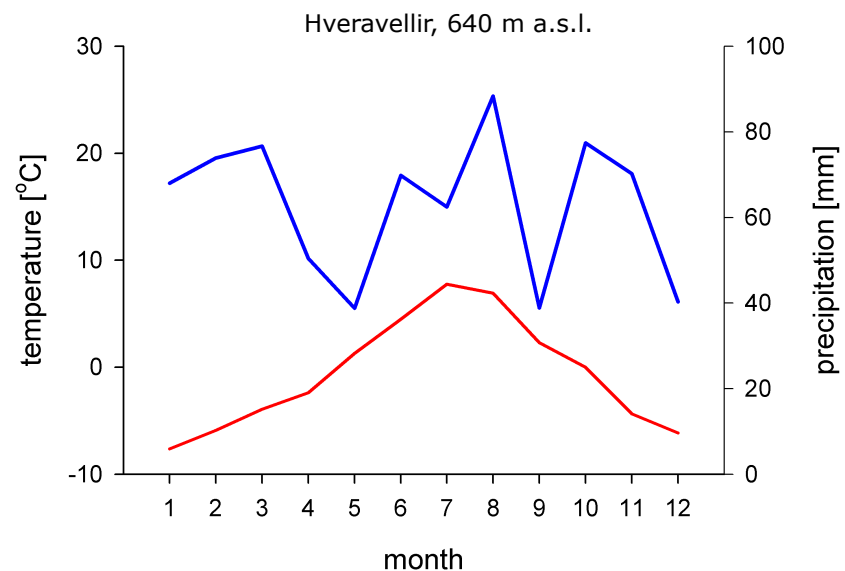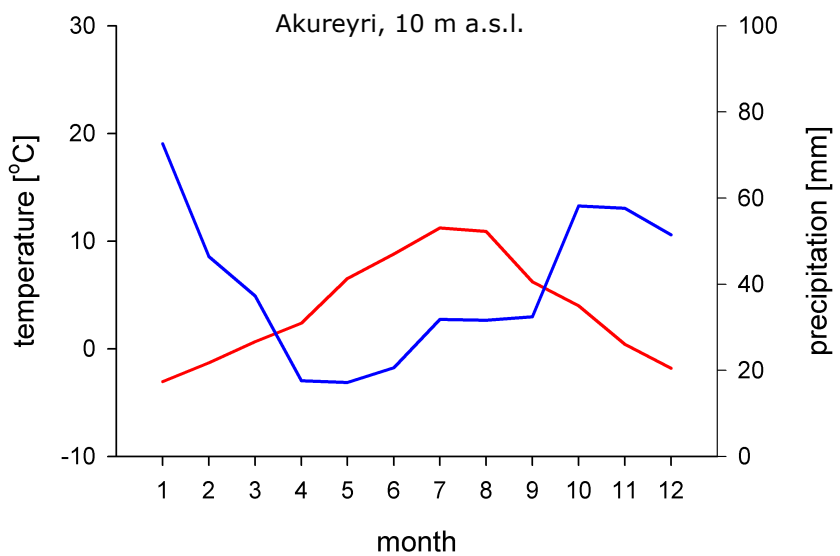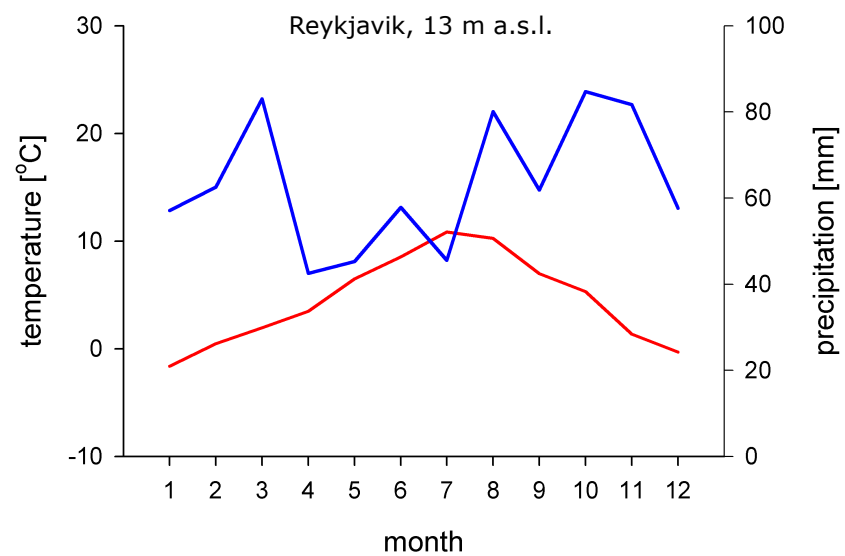

Supplement: Supplemental Information 1 [file peerj-04-1559-s001.pdf]
